# Beyond socioeconomic status: the cross-cultural interplay of perceived teacher social support in reading literacy

**DOI:** 10.3389/fpsyg.2025.1642504

**Published:** 2025-11-11

**Authors:** Shuai Xu, Ping Yong

**Affiliations:** 1 School of Marxism, Sias University, Zhengzhou, China; 2 College of International Education, Sichuan International Studies University, Chongqing, China; 3 Graduate School of Education, Sehan University, Yeongam, Republic of Korea

**Keywords:** socioeconomic status, teacher social support, reading literacy, Cultural Value Orientation Theory, PISA 2022

## Abstract

Previous research has extensively examined the relationship between student socioeconomic status (SES) and their reading literacy. However, few studies have explored how the different facets of perceived teacher social support (TSS) moderate the SES-reading literacy link, particularly from a cross-cultural perspective. The present study utilized data from the 2022 Programme for International Student Assessment, which encompasses 515,170 students across 70 countries and economies. These countries/economies were grouped into eight distinct cultural clusters based on Cultural Value Orientation Theory: African and Middle Eastern, Confucian, East-Central European, East European, English-speaking, Latin American, South Eastern, and West European cultures. Employing structural equation modeling, we found a consistent positive correlation between SES and reading literacy across all eight cultural contexts. Distinct TSS facets (i.e., teacher support, teacher emotional support, and teacher feedback) exhibited varying moderating effects on the SES-reading literacy relationship across different cultures. Furthermore, the variations in effect size across the eight cultural clusters are explicable by cultural values. Our study underscores the necessity of differentiating the TSS facets and the importance of cultural context in assessing the interactions among the investigated variables.

## 1 Introduction

In previous decades, Socioeconomic Status (SES) has been identified as a key determinant of students’ academic performance, as evidenced by several seminal works (Coleman, 1966; Harwell et al., 2021; Sirin, 2005). A comprehensive meta-analysis has found that SES has a significant impact on student achievement (Janssen et al., 2019). Nevertheless, the magnitude of this impact exhibits variability across different contexts and countries, warranting a more rigorous and nuanced exploration.

Extant literature posits Teacher Social Support (TSS) as a potential moderating factor in the nexus between SES and academic outcomes. Cultural reproduction theory (CRT; Bourdieu, 1973, 1986) posits that educational systems inherently perpetuate existing social hierarchies by validating the cultural capital that is more commonly found among high-SES students. This often leads to better academic outcomes for these students, reinforcing social inequalities (Byun et al., 2012; Puzić et al., 2016; Tan, 2015). In contrast, several studies have claimed that high SES students report lower TSS than low SES students (Atlay et al., 2019). This paradox may be attributed to the overlooked multidimensional nature of TSS. TSS is a multifaceted concept that encapsulates various dimensions, including teacher support, teacher emotional support, and teacher feedback (Bakchich et al., 2023; Tao et al., 2022; Wentzel et al., 2010). Yet, research has not empirically dissected how the multifaceted dimensions of TSS might intricately modulate the relationship between SES and academic outcomes.

Moreover, prior studies have neglected the role of cultural factors. Cultural norms and values significantly shape educational expectations (Li and Xie, 2020), attitudes (Benoliel and Berkovich, 2018), and interpretations of social capital (Scrivens and Smith, 2013), which alter the magnitude of the influence of SES and TSS (Liu et al., 2020; Schwartz, 2009). Understanding cultural specificity is essential for a more comprehensive exploration of the dynamics among SES, TSS, and student outcomes, especially when considering the global context of education.

To address these gaps, namely the often-neglected multidimensional nature of TSS and the overlooked influence of cultural variables, this study utilizes data from the 2022 Program for International Student Assessment (PISA). Specifically, based on Bourdieu’s (1973, 1986) CRT, we investigate the moderating role of three TSS facets (i.e., teacher support, teacher emotional support, and teacher feedback) in the relationship between SES and student reading achievement across eight distinct cultural groups: African and the Middle Eastern, Confucian, East-Central European, East European, English-speaking, Latin American, South Eastern, and West European (Schwartz, 2009). Specifically, we explored following questions:

1. To what extent does student SES serve as a predictor for student reading literacy across Schwartz’s eight cultures?
2. How does three TSS facets moderate the link between student SES and reading literacy cross eight cultures?

In this study, we expand TSS literature by building on studies that consider the multifaceted nature of TSS in relation to academic outcomes (Bakchich et al., 2023). We further build on the multicultural education literature by applying Schwartz’s (2009) cultural framework as a lens through which to scrutinize the complex relationships among SES, TSS, and reading literacy.

## 2 Literature review

### 2.1 SES and student achievement

The SES refers to an individual or family’s social and economic standing regarding society’s income (Batruch et al., 2017; Jury et al., 2017). It is determined by multiple factors, including parental education level, occupation, income, and access to resources and opportunities (Mistry et al., 2010). Coleman (1966) conducted a large-scale survey involving 640,000 students across 4,000 schools in the United States, and published the famous Coleman Report. The seminal study concluded that family SES has the most substantial influence on students’ academic performance, overshadowing the role of schools.

As a large international assessment, the PISA 2022 results indicated that students with socioeconomic advantages typically outperform their disadvantaged counterparts in reading literacy (OECD, 2023). One contributing factor may be out-of-school English exposure, such as watching videos or reading print (Tsang and Lo, 2025). Such informal exposure, modestly linked to SES indicators like income and paternal education, predicts vocabulary growth, which is a core component of reading proficiency. However, the size of this performance gap varies significantly among countries. In 11 OECD members (e.g., Canada, Finland, and the United Kingdom), the average performance exceeded the OECD mean; however, the correlation between SES and reading achievement was less pronounced than the OECD average. This implies that the strength of SES as a predictor of reading literacy may vary among students.

### 2.2 Teacher social support

Social support is defined as the perceived assistance from one’s social network (Malecki and Demaray, 2023). Research highlights parents, teachers, and peers as primary support sources for students. However, this study focuses on Teacher Social Support (TSS) for its unique impact: teachers’ authoritative roles allow them to influence classroom dynamics (Keiler, 2018), and their pedagogical knowledge supports the implementation of student-centered interventions (Ertmer and Ottenbreit-Leftwich, 2005).

The TSS serves as a pivotal factor influencing student success and development within educational settings (Eisenberger et al., 2020). Regarding the relationship between TSS and student achievement, Tao et al. (2022) have conducted a meta-analysis of 71 empirical studies. They found that, despite the contradictory TSS impact on student academic outcomes, a modest yet statistically significant correlation was identified, quantified by a correlation coefficient of *r* = 0.16. However, their study also underscored the need to conceptualize TSS as a multidimensional construct because various nomenclatures and categorizations have been used, such as emotional, informational, and instrumental support (Tardy, 2019), as well as emotional and academic support (Patrick et al., 2010). Given its inherent multidimensionality, this fragmentation of TSS hampers coherent theoretical development and empirical investigations (Bakchich et al., 2023). Thus, to provide a more comprehensive understanding of TSS, this study uses a multidimensional conceptualization of TSS in PISA 2022, which includes teacher support (TS), teacher emotional support (TES), and teacher feedback (TF; Atlay et al., 2019; OECD, 2023).

The TS has been defined as the care and quality of relationships teachers have with their students (Hoferichter et al., 2022). TS positively affects students who encounter academic challenges by enhancing their sense of control over their learning outcomes and increasing their engagement in the educational process, which, in turn, boosts their reading performance (Tao et al., 2022). TES is a feature of teacher-student interactions that enhances both students’ emotional well-being and academic performance (Eisenberger et al., 2020). Students feel emotionally supported in school when they are treated with fairness and respect by their teachers, sense warm and non-judgmental attitudes from teachers, and feel that their ideas are heard and valued (Zhan et al., 2023). TES has been consistently identified as a strong indicator of key educational outcomes, including the development of academic and social outcomes (Saeed et al., 2021). TF is the guidance or comments provided by teachers to help students improve their learning by altering their thought processes or behaviors. A meta-analysis has revealed a medium effect (*r* = 0.28) of feedback on student learning (Wisniewski et al., 2020).

### 2.3 Teacher social support as a moderator

Cultural reproduction theory (Bourdieu, 1973, 1986) suggests that TSS may exaggerate the relationship between student SES and achievement. Compared to low SES parents, higher SES parents are more inclined to engage in activities that align with the values of the education system (e.g., frequenting museums or fostering reading habits; Bakchich et al., 2023) and educate their children in a manner that aligns with the expected norms of teacher-student interactions (Sortkær, 2019). Consequently, students from high SES backgrounds are more likely to engage with their teachers on an equal footing, while those from low SES backgrounds tend to maintain more hierarchical and distant relationships with their teachers (Calarco, 2014). Therefore, the positive outcomes associated with SES, such as enhanced student achievement, are more likely to be realized by students receiving more TSS than those receiving less TSS.

However, several studies have challenged the validity of CRT’s claims. For example, OECD (2019) has reported a negative link between TSS and SES, particularly regarding the relevance of TS (Bakchich et al., 2023). This may be because high SES students are more critical of their teachers than low SES students (Atlay et al., 2019) or because low SES students, who may have lower expectations for teacher academic support, tend to assess such support positively even when it is minimal (Bakchich et al., 2023).

In contrast to CRT, which focuses on cultural reproduction through social capital, Self-Determination Theory (SDT) offers a complementary perspective by emphasizing how supportive teacher behaviors fulfill students’ psychological needs. Students from different SES backgrounds may perceive and benefit from TSS differently depending on whether the support addresses their need for relatedness, competence, or autonomy needs that may be more or less salient in various SES and cultural contexts (Domen et al., 2020). This perspective helps explain the inconsistent or even negative associations between TSS and SES found in previous studies. Therefore, a following question arises: Do all three TSS facets have a positive moderating effect on the link between SES and student achievement?

### 2.4 Schwartz’s cultural values

In a cross-national study encompassing 36 countries, Baker et al. (2002) has found that student SES accounts for 1.5–20% of the variance in math and science test scores. Chiu (2010) has reported similar findings based on information from participants from 41 countries. However, these cross-cultural studies endeavor to investigate the impact of a country’s economic development level (e.g., Gross National Product) on educational outcomes. For instance, the Heyneman-Loxley effect posits that in countries with lower income levels, school-level variables may account for a more substantial proportion of the variance in student achievement compared to individual-level characteristics.

**Figure 1:**
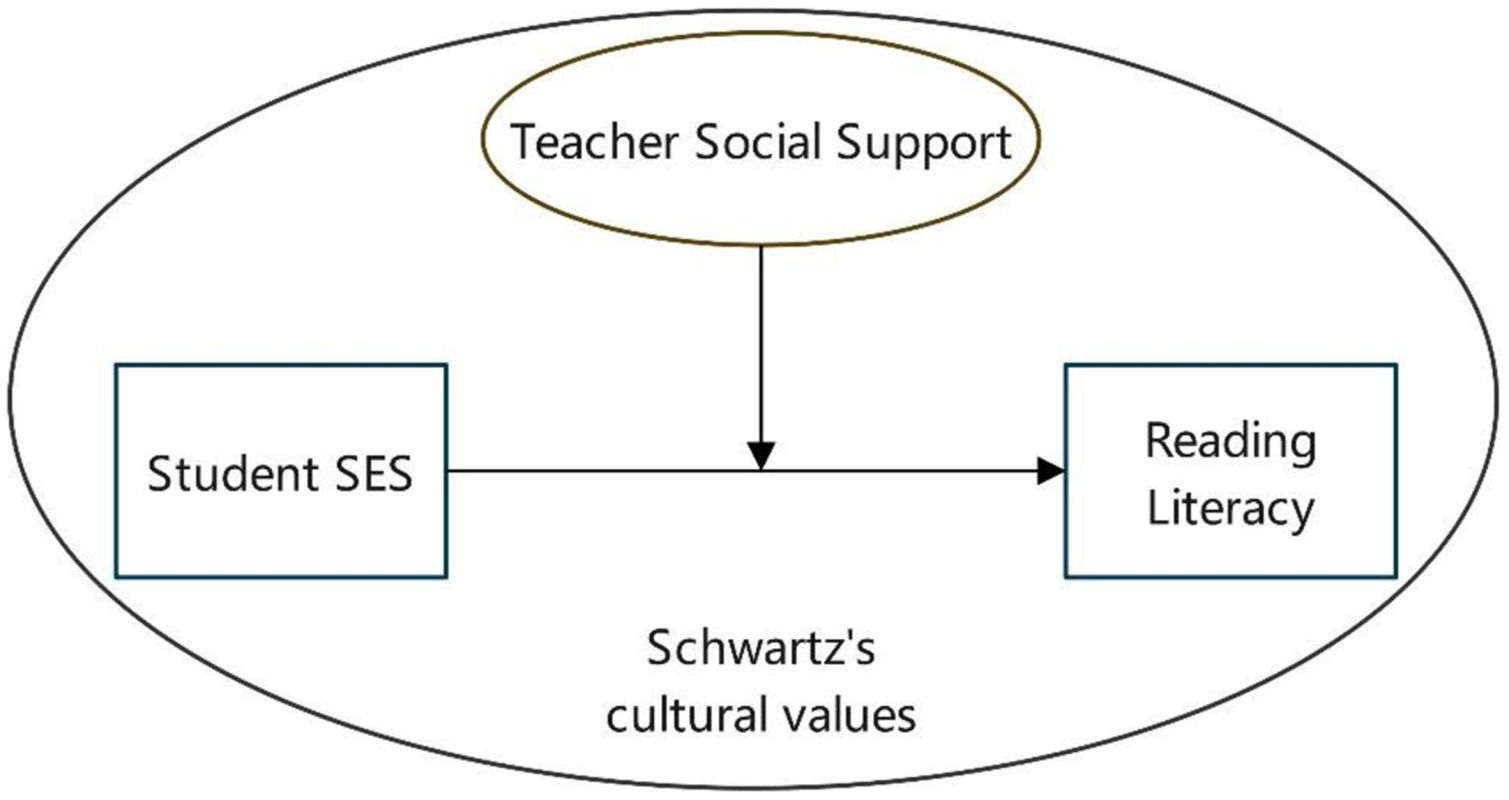
Conceptual model.

We argue that country-level cultural values are another factor meriting further attention. According to Expectancy-value theory (EVT), individuals’ choices and persistence in education are shaped by their expectations and the value they place on their tasks (Wigfield et al., 2009). These beliefs are influenced by personal goals, self-perceived abilities, and task difficulty, which are further shaped by the opinions and attitudes of key social figures, such as parents and teachers (Goodnow, 1988). Cultural context also plays a role in shaping these social influences and, in turn, affects children’s perceptions and beliefs, including their interpretations of TSS and social capital. Li and Xie (2020) discovered that in East Asian societies, educational expectations are less influenced by family background than they are in Western societies. Lo et al. (2024) provide micro-level evidence from a cross-cultural study comparing students in Hong Kong and the United Kingdom, highlighting systematic differences in independent learning strategies. Hong Kong students tend to emphasize rehearsal, organization, and time management, whereas UK students report stronger self-regulation, critical thinking, and self-efficacy. These findings suggest that hierarchical and embedded cultural value orientations promote externally structured learning habits, making teacher structuring particularly beneficial for students who depend on guided planning and access to learning resources (Figure 1).

**Figure 2:**
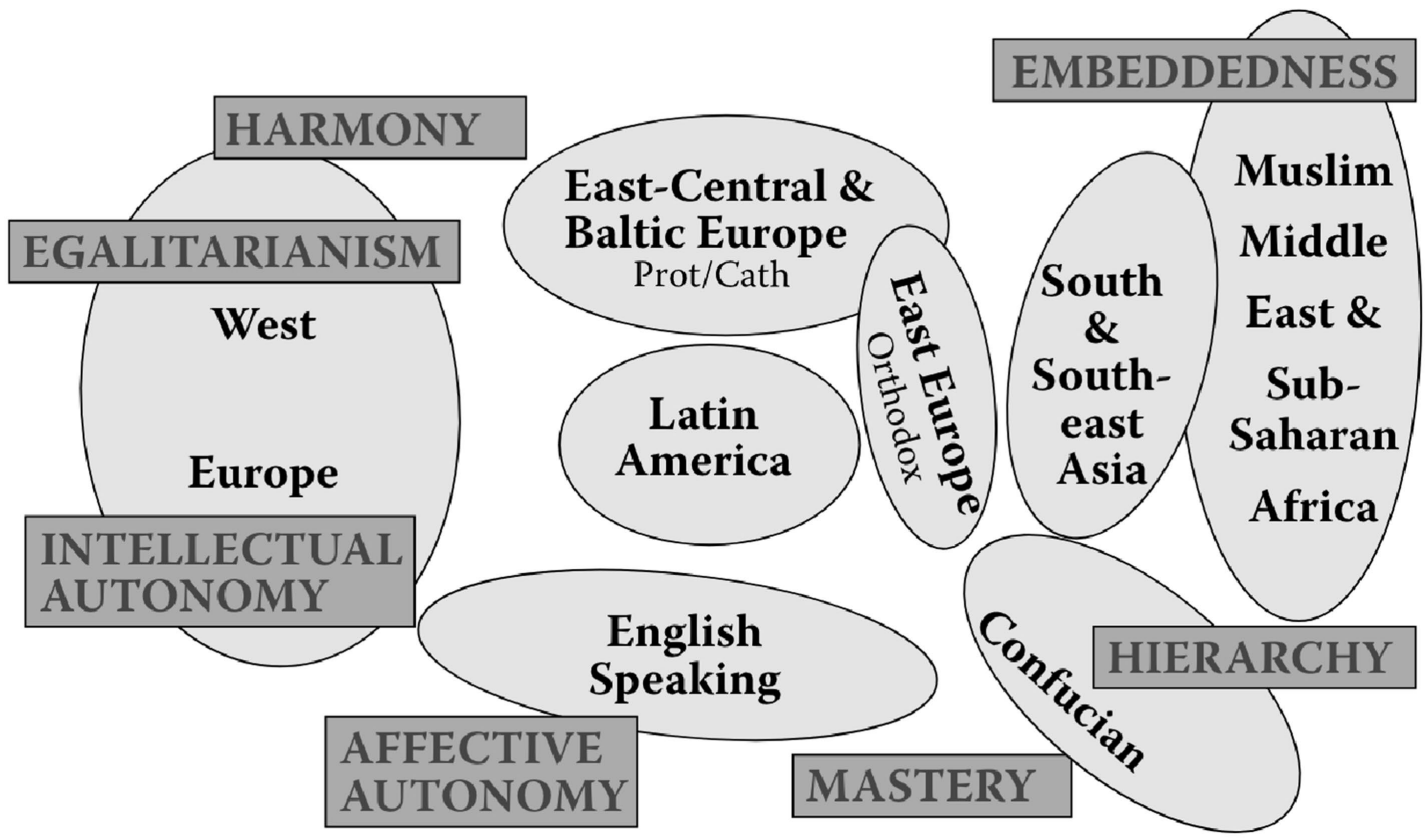
A typology of Schwartz’s (2009) cultural groups. This figure was adapted from “Culture Matters” produced by Schwartz (2009, p. 136). Reprinted with permission.

However, it would be reductive and hasty if we divide culture into these two categories. Schwartz’s (2009) Cultural Value Orientation Theory proposes three core dimensions that differentiate cultures: autonomy versus embeddedness, hierarchy versus egalitarianism, and mastery versus harmony (Figure 2). The first pair explores whether people feel more connected with groups or seek personal independence; the second delves into societal norms and questions whether people accept or challenge power dynamics; and the third focuses on how people interact with the world, whether by striving to control or harmonize with it.

Drawing on these cultural dimensions, Schwartz (2009) categorized various countries into eight distinct cultural clusters: Western European, Eastern Central European, Eastern European, Latin American, English-speaking, Confucian, Southeast Asian, and African and the Middle Eastern. In particular, Western European cultures prioritize autonomy but also value egalitarianism and harmony, diverging from traditional individualistic narratives. Confucian and South Asian cultures emphasize hierarchy but differ in secondary values such as mastery and embeddedness, respectively. Eastern Central European cultures displays higher harmony and intellectual autonomy but a lower hierarchy than Eastern European cultures. Latin American cultures are characterized by high hierarchy and embeddedness but low intellectual autonomy. The English-speaking cultural regions focus on autonomy and mastery. Confucian culture underscores hierarchy, embeddedness, and mastery. Middle Eastern and African cultures prioritize social relationships (high embeddedness) over individualism (low autonomy). Finally, Southeast Asian cultures emphasize the fulfillment of obligations within hierarchical systems.

Schwartz’s cultural value theory has been widely applied across interdisciplinary fields, influencing research in areas such as consumer behavior (Jahandideh et al., 2014), earnings management (Desender et al., 2011), financial policy (Shao et al., 2010), and personal health status (Akaliyski et al., 2022). In the field of education, previous studies have investigated the influence of cultural values on information and communication technology diffusion levels (Choden et al., 2019) and academic performance (Benoliel and Berkovich, 2018). It has also been employed to examine how the relationship between needs-supportive teaching methods, student achievement, and well-being varies across cultural contexts (Haw and King, 2022; Wang et al., 2021). However, the extant literature has yet to offer a comprehensive examination of the moderating role of TSS in the relationship between SES and student achievement, particularly regarding its consistency across diverse cultural groups.

### 2.5 Differential impacts of three TSS facets across cultures

Applying the cultural framework of Schwartz (2009) to our study, we propose that each facet of teacher support addresses distinct psychological needs, which are valued differently across cultures. TS, which revolves around the care and quality of relationships teachers establish with students, is likely to manifest differently in cultures that prioritize hierarchy versus those that value egalitarianism. In hierarchical cultures, such as many found in East Asia and parts of Eastern Europe, TS is expected to be more authoritative and structured, reflecting the societal emphasis on respect for authority and tradition. This form of support is geared toward maintaining discipline and promoting a structured learning environment. Conversely, in egalitarian cultures, such as those in Scandinavia, TS tends to be more democratic and inclusive, aiming to empower students by treating them as equals and encouraging participatory learning. This approach fosters an educational atmosphere where students feel more engaged and valued, potentially increasing their motivation and academic achievement. From the SDT perspective, TS supports students’ need for competence by providing structured guidance and academic scaffolding. In hierarchical cultures, such support may be perceived as authoritative yet constructive, reinforcing students’ sense of mastery (King et al., 2024). In contrast, in egalitarian cultures, TS may promote competence by encouraging students to take ownership of their learning through more democratic teacher-student interactions. Beyond classroom interactions, TS may also guide students, especially those from lower-SES backgrounds, toward structured, low-cost exposure, such as subtitled videos or graded readers. This aligns with Tsang and Lo (2025), who found that informal English input predicts vocabulary growth. TS may therefore support both in-school learning and out-of-school engagement.

The TES focuses on fostering students’ emotional well-being and is critical in cultures that emphasize embeddedness and affective autonomy. In cultures with strong embeddedness values, such as many Middle Eastern and Latin American societies, TES is integral to the educational experience, with a significant emphasis placed on creating a nurturing, family-like classroom environment. This form of support helps in building strong emotional bonds between students and teachers, enhancing students’ sense of security and belonging. On the other hand, in cultures that highlight affective autonomy, like many Western countries, TES might involve supporting students’ emotional independence and self-expression, helping them to develop resilience and personal coping strategies in the academic setting. As conceptualized in SDT, TES primarily fulfills students’ basic psychological need for relatedness, which refers to feeling connected and valued by others (Escandell and Chu, 2023). In embedded cultures, this need is met through emotionally expressive teacher-student relationships. In contrast, in cultures that emphasize affective autonomy, relatedness may be expressed through mutual respect and support for emotional independence, showing that TES can remain effective across cultural contexts, albeit in different forms.

TF varies significantly between cultures that value mastery compared to those that prioritize harmony. In mastery-oriented cultures, such as the United States and parts of Western Europe, TF is typically direct and explicit, aimed at improving individual performance and fostering personal achievement. In contrast, in harmony-oriented cultures, such as many Southeast Asian countries, TF is likely to be more subtle and indirect, intended to preserve interpersonal harmony and avoid direct confrontation. In these settings, feedback is often integrated with positive reinforcement to ensure that it is received constructively, supporting a cohesive learning environment without causing distress or embarrassment to students.

Few studies have specifically examined how these TSS facets influence reading performance across diverse cultural settings, highlighting a gap in the literature. The need for distinct hypotheses regarding the effectiveness of TS, TES, and TF in different cultural contexts is clear. By distinguishing between these facets and their culturally contingent impacts, this study aims to contribute to a nuanced understanding of how teacher support can be optimized across diverse educational settings.

## 3 Method

### 3.1 Sample

The present study constitutes a secondary analysis of the PISA 2022 (OECD, 2023). This program aims to globally evaluate the competencies of 15-year-old students to prepare them for future challenges. In 2022, the PISA team focused on the reading literacy of students. Hence, this study focuses on reading-related variables. Data encompassing 515,170 students from 70 countries were utilized, with countries and economies being segmented into eight cultural groupings (Schwartz, 2009; Wang et al., 2021). Table 1 reports the sample countries in each culture group, sample size, and percentage of women. Across the eight cultures, the sample size ranged from 24,233 to 112,253, and the proportion of female participants fluctuated from 48.6 to 52.1%.

**Table 1:** Descriptive statistics of each cultural group (*N* = 515,170).

Culture	Country/economies	Students (n)	Women (%)
African and Middle Eastern	Jordan, Morocco, Qatar, Saudi Arabia, Turkey, United Arab Emirates	61,908	49.4%
Confucian	Korea, Japan, Macau, Hong Kong, Chinese Taipei	41,872	49.0%
East-Central European	Albania, Croatia, Czech Republic, Estonia, Hungary, Kosovo, Latvia, Lithuania, Poland, Romania, Slovak Republic, Slovenia	70,747	49.6%
East European	Azerbaijan, Belarus, Bosnia and Herzegovina, Bulgaria, Georgia, Kazakhstan, Moldova, Moscow Region (RUS), Russia, Serbia, Tatarstan (RUS), Ukraine	81,955	48.6%
English Speaking	Australia, Canada, Ireland, United Kingdom, United States	24,233	50.1%
Latin American	Argentina, Brazil, Uruguay, Chile, Colombia, Costa Rica, Dominican Republic, Mexico, Panama, Peru	75,922	51.1%
South East Asian	Brunei Darussalam, Indonesia, Malaysia, Philippines, Thailand, Vietnam	46,280	52.1%
West European	Austria, Greece, Iceland, Italy, Luxembourg, Malta, Netherlands, Portugal, Spain, Sweden, Switzerland, Finland, France, Germany	112,253	49.2%

### 3.2 Measures

#### 3.2.1 Independent variable

PISA 2022 assessed student SES through an index of economic, social, and cultural status (ESCS), which is a composite indicator aggregating the economic, social, cultural, and human capital resources accessible to students into a unified score (Harris, 2023).

#### 3.2.2 Moderator

The PISA 2022 measured three key TSS facets: TS, TES, and TF (Bakchich et al., 2023). While there has been limited empirical investigation into TES and TF, previous scholarly contributions have examined the constructs related to these variables (Malecki and Demaray, 2023). These three facets are substantiated in the literature as pivotal factors in fostering constructive teacher-student relationships and are linked to significant outcomes such as student learning, engagement, and achievement (Eisenberger et al., 2020).

TS was measured by four items. An example is “The teacher shows an interest in every student’s learning,” with a rating scale ranging from 1 = “Every lesson” to 4 = “Never or hardly ever.” TES was measured using three items. A sample item is “The teacher made me feel confident in my ability to do well in the course,” rated from 1 = “Strongly disagree” to 4 = “Strongly agree.” TF was measured using three items. An example item is “The teacher gives me feedback on my strengths in this subject,” rated from 1 = “Never or hardly ever” to 4 = “Every lesson or almost every lesson.”

#### 3.2.3 Dependent variables

Our study focuses on reading achievement, as it tends to be more susceptible to sociocultural and national influences compared to mathematics and science achievement. However, The PISA 2022 did not provide direct scores for student achievement; instead, it presented 10 plausible values for each student. Each plausible value was randomly drawn from the posterior distribution of the student scores. Technically, to ensure accuracy, plausible values were calculated using item response theory to control for measurement errors and regression modeling to control for errors due to contextual factors (Harris, 2023). To ensure unbiased parameter estimates and appropriately reflect uncertainty, we followed the recommended procedure from the PISA technical documentation by analyzing each of the 10 plausible values separately and combining the results using Rubin’s Rules (Huang, 2024). This approach pools the point estimates and adjusts standard errors to account for both within- and between-imputation variance, thus providing more accurate and conservative inference. All plausible values were first standardized using the international mean and standard deviation, allowing interpretation of results in standard deviation units relative to the global student average.

For the above variables, we recoded the TS items so that higher values indicate greater perceived support. This recoding ensures that, across all key variables, higher values consistently represent greater levels of the underlying construct (e.g., higher SES, stronger support, better reading achievement), thereby facilitating consistent interpretation across variables.

#### 3.2.4 Covariates

Individual- and school-level covariates were included to control for confounding factors. Individual-level variables included gender (coded as 0 = woman vs. 1 = man), immigration status (coded as 0 = native, 1 = second generation, and 2 = first generation), parental emotional support, and disciplinary climate, all of which are related to reading literacy (Chen et al., 2022; Rvachew et al., 2020). Parental emotional support reflecting parents’ involvement in their child’s well-being and a emotionally supportive family environment. It was assessed using three items from the PISA Parental Emotional Support scale (e.g., “My parents encourage me to be confident”), with good internal consistency with Cronbach’s *α* ranging from 0.72 to 0.85 across cultures. Disciplinary climate is defined as a measure of the extent to which students experience disruptions in their learning environment. It was measured using five items (e.g., “How often: Students cannot work well”), with higher scores reflecting more orderly classrooms; reliability ranged from 0.72 to 0.83 across cultural group.

We also included school type (coded as 1 = Private independent, 2 = Private Government-dependent, 3 = Public), location (ranging from 1 = a village or rural area with fewer than 3,000 people to 6 = a megacity with over 10 million people), and size as covariates (Kim et al., 2018).

In our dataset, less than 6% of the data per case were missing for the variables under study. Thus, we employed Markov Chain Monte Carlo techniques for multiple imputations of the missing data in the *mice* package in R (Van Buuren and Groothuis-Oudshoorn, 2011). Ten imputed datasets were generated. The imputation model included all covariates used in the analysis, but excluded the independent variable, moderator, and dependent variable to avoid bias in estimating relationships among key constructs.

### 3.3 Measurement model

Prior to the main analysis, we examined the invariance of the three TSS facets measurement model across the eight cultures. Each of the three TSS facets was modeled as a latent factor with its original item indicators. Model fit was evaluated using the Comparative Fit Index (CFI), Tucker-Lewis Index (TLI), and Root Mean Squared Error of Approximation (RMSEA). Standards for acceptable fit were defined by a CFI and TLI greater than 0.90 and an RMSEA less than 0.08, in line with the existing literature (Hu and Bentler, 1999). Furthermore, we adopted specific thresholds for assessing metric and scalar invariance (ΔCFI ≤ 0.010, ΔRMSEA ≤ 0.015; Chen, 2007).

### 3.4 Statistical analyses

Given the complexity of the relationships among SES, TSS, and academic achievement, especially when considering cultural moderation, Structural Equation Modeling (SEM) was deemed the most appropriate method for this study. SEM allows the simultaneous estimation of multiple interrelated relationships, providing a more comprehensive understanding of the mechanisms at play. Unlike more traditional linear regression models, SEM enables the examination of both direct and indirect effects, thereby offering a nuanced approach for assessing how TSS moderates the relationship between SES and academic achievement.

Because the three TSS facets are each measured by multiple items while SES is an observed composite in PISA 2022, moderation was specified as latent-by-observed interactions. Product indicators were generated by multiplying the mean-centered observed SES score with each item indicator of the respective TSS factor (using semTools:indProd), and these products served as manifest indicators of the interaction latent factors. This strategy preserves measurement-error information for the TSS facets while directly using the observed SES indicator provided by PISA.

To account for the hierarchical structure of the PISA 2022 dataset, we incorporated weights and replicates into our statistical analyses (Haw and King, 2022; Huang, 2016; McNeish et al., 2017), instead of multilevel modeling.^1^ Using Anderson and Gerbing’s (1988) two-step methodology for SEM, all SEM computations were performed using the *lavaan* package (Rosseel and Rosseel, 2012); product indicators for latent-by-observed interactions were created with semTools:indProd, and survey-adjusted standard errors and test statistics were obtained with lavaan.survey using a pooled within-cluster covariance approach following Oberski (2014). Prior studies have shown that, when the focus is on student-level latent constructs, and when design-based corrections (e.g., cluster-adjusted standard errors, sampling weights) are applied, single-level SEM can yield unbiased and interpretable parameter estimates (Oberski, 2014). Therefore, we employed a pooled within-cluster covariance matrix to adjust for school-level clustering. Given that our analysis grouped countries into broader eight cultural clusters, but PISA replicate weights are constructed at the national level, we retained the original country-specific replicate weights within each cultural group. We acknowledge that this approach does not reconstruct replicate weights at the cultural-cluster level, which may introduce limitations in fully aligning sampling variance estimation with the pooled analysis.

Initially, we conducted Confirmatory Factor Analysis (CFA) for each culture. After evaluating the model-data fit, we proceeded to apply our extensive structural model. During both stages, we refined our model by employing a pooled within-cluster covariance matrix to account for the sampling structure (Oberski, 2014). The fit of the structural model was assessed using the same fit index criteria as those employed in the CFA. Models were estimated using robust maximum likelihood (MLR) to obtain robust standard errors and test statistics (Byrne and de Vijver, 2017). Data cleaning and statistical analyses were conducted using R version 4.0.3 (R Core Team, 2020).

## 4 Results

### 4.1 Descriptive statistics

Table 2 displays the descriptive statistics and bivariate correlations of the overall sample. The results show that most bivariate correlations are significant. Among the independent (SES), moderator (TS, TES, TF), and dependent (reading literacy) variables, all bivariate correlations for these variables yield a *p*-value less than 0.05, indicating statistical significance. The uniform significance of these variables suggests intricate relationships among them.

**Table 2:** Descriptive statistics and correlation among the variables for the overall sample.

		1	2	3	4	5	6	7	8	9	10	11
1	Student SES	—										
2	Gender	0.01	—									
3	Immigration	**0.05**	0	—								
4	Parental emotional support	**0.14**	**−0.08**	0.01	—							
5	Disciplinary climate	**0.04**	−0.03	−0.01	**0.11**	—						
6	School type	**0.19**	0.01	**0.16**	**0.04**	0.01	—					
7	School location	**0.2**	0.01	**0.13**	0.02	0.01	**0.22**	—				
8	Teacher support*	**0.17**	−0.01	0	**0.15**	**0.17**	0.02	−0.02	—			
9	Teacher emotional support	0.03	**−0.04**	0	**0.19**	**0.21**	0.03	−0.02	**0.36**	—		
10	Teacher feedback	−0.02	**0.06**	**0.04**	**0.14**	**0.18**	0.02	−0.01	**0.37**	**0.4**	—	
11	Reading literacy	**0.41**	**−0.14**	0.02	**0.17**	**0.12**	**0.13**	**0.19**	**0.12**	**0.13**	**0.19**	
Descriptive statistics												
	Mean	0.28	1.5	0.19	−0.03	2.65	1.2	3.21	3.2	2.82	2.38	0
	SD	1.12	0.5	0.52	1	0.78	0.4	1.2	0.79	0.77	0.85	1
	Min	−8.17	1	1	−2.45	1	1	1	1	1	1	−3.67
	Max	4.21	2	2	1.03	4	2	5	4	4	4	3.49

SES, socioeconomic status; SD, standard deviation. Gender (1 = woman, 2 = man), immigration status (1 = native, 2 = immigrant), school type (1 = public, 2 = private), and school location (e.g., rural); *: the items measuring teacher support were all reversely recoded; bold indicates a significant correlation at *p* < 0.05.

### 4.2 Measurement invariance

The results in Table 3 depict a sequence of models testing measurement invariance. The Configural Invariance model establishes a good baseline fit (CFI = 0.983, TLI = 0.978, RMSEA = 0.038, and SRMR = 0.023), signifying the same underlying structure across the groups. The Metric Invariance model demonstrates equivalence in factor loadings (CFI = 0.980, TLI = 0.978, RMSEA = 0.038, SRMR = 0.027, ΔCFI = <0.002, ΔRMSEA < 0.001, and ΔSRMR<0.001), with negligible changes in fit. The Scalar Invariance model shows a substantial change (CFI = 0.959, TLI = 0.957, RMSEA = 0.053, SRMR = 0.034, ΔCFI = 0.022, ΔRMSEA = 0.014, and ΔSRMR = 0.009), indicating non-equivalence in intercepts across groups. Guided by Steenkamp and Baumgartner’s (1998) methodology, we eased the equality constraints on two parameters for TS and one parameter for TES; at least two parameters were constrained to be equal for each TSS facet (see Appendix for more details). The model then demonstrates an acceptable fit (CFI = 0.979, TLI = 0.978, RMSEA = 0.037, SRMR = 0.025, ΔCFI = 0.009, ΔRMSEA = 0.011, and ΔSRMR = 0.008), suggesting that comparisons of the latent construct across groups are plausible, with caution relating to the invariance of certain intercepts. Appendix Table S1 further examined factor loading ranges and reliability indices (Cronbach’s *α* and McDonald’s *ω*) of each TSS facet.

**Table 3:** Model fit statistics for teacher social support across cultures.

Model	*χ* ^2^	df	CFI	TLI	RMSEA	SRMR	∆CFI	∆RMSEA
African and Middle Eastern								
M1	11701.67	130	0.91	0.90	0.08	0.06		
M2	12832.18	165	0.9	0.90	0.08	0.07	0.01	0.01
M3	15864.88	200	0.88	0.87	0.09	0.07	0.02	0.01
M4	12937.82	180	0.9	0.89	0.08	0.06	0.01	0.01
Confucian								
M1	2857.27	110	0.96	0.95	0.06	0.04		
M2	3618.05	138	0.95	0.95	0.06	0.05	0.01	0.00
M3	7421.91	166	0.93	0.92	0.07	0.06	0.02	0.01
M4	4506.63	149	0.94	0.94	0.06	0.05	0.00	0.01
East Central European								
M1	5125.77	230	0.97	0.95	0.06	0.04		
M2	6121.92	296	0.97	0.95	0.06	0.05	0.00	0.01
M3	9894.05	362	0.95	0.94	0.07	0.06	0.02	0.01
M4	7185.91	318	0.96	0.95	0.06	0.05	0.01	0.01
East European								
M1	9787.25	245	0.95	0.93	0.07	0.05		
M2	10809.61	320	0.94	0.93	0.07	0.06	0.00	0.01
M3	15001.11	395	0.93	0.92	0.09	0.07	0.01	0.02
M4	11829.07	350	0.94	0.93	0.07	0.06	0.00	0.01
English Speaking								
M1	1416.15	90	0.98	0.98	0.04	0.02		
M2	1452.93	114	0.98	0.98	0.04	0.02	0.00	0.00
M3	1671.58	138	0.95	0.97	0.05	0.03	0.03	0.01
M4	1508.44	124	0.97	0.98	0.04	0.02	0.01	0.00
Latin American								
M1	6112.55	190	0.96	0.95	0.06	0.04		
M2	6367.73	245	0.95	0.95	0.06	0.04	0.00	0.01
M3	8957.62	300	0.93	0.94	0.07	0.05	0.02	0.01
M4	7269.48	270	0.94	0.94	0.07	0.05	0.01	0.01
South East Asian								
M1	4945.16	130	0.94	0.93	0.06	0.04		
M2	6243.73	160	0.93	0.93	0.07	0.05	0.01	0.01
M3	11944.38	190	0.91	0.9	0.08	0.06	0.02	0.01
M4	6699.36	175	0.93	0.93	0.07	0.05	0.01	0.01
West European								
M1	4504.03	307	0.98	0.98	0.04	0.03		
M2	6534.83	398	0.98	0.97	0.04	0.04	0.00	0.00
M3	9033.84	489	0.95	0.97	0.06	0.04	0.03	0.02
M4	7216.74	434	0.97	0.97	0.05	0.04	0.01	0.01
Overall sample								
M1	46015.89	568	0.98	0.978	0.038	0.023		
M2	52504.66	638	0.98	0.978	0.038	0.027	0.00	0.00
M3	109838.4	708	0.96	0.957	0.053	0.034	0.02	0.01
M4	75064.15	672	0.98	0.978	0.037	0.025	0.01	0.01

M1 = configural invariance, M2 = metric invariance, M3 = scalar invariance, M4 = partial scalar invariance. df: degrees of freedom. CFI: Comparative Fit Index; TLI: Tucker-Lewis Index; RMSEA: Root Mean Squared Error of Approximation; SRMR: Standardized Root Mean Square Residual. Partial scalar invariance is achieved for each culture and the overall sample.

### 4.3 SEM

The SEM results are presented and summarized in Tables 4, 5, respectively. The top half of Table 4 shows that SEM had an acceptable fit across the eight cultural groups. However, the English-speaking culture was an exception, with a CFI of 0.90 and a TLI of 0.89. However, these indices still fall within the marginally acceptable range (e.g., CFI ≥ 0.90; RMSEA ≤ 0.08) as suggested by previous literature (Marsh et al., 2004). Given the complexity of the model, the inclusion of latent constructs, and the cross-cultural nature of the data, such marginal fit can be expected and is considered acceptable in large-scale international studies (Byrne and de Vijver, 2017). Therefore, we interpret the model as adequately fitting for the English-speaking sample.

**Table 4:** Relationships among socioeconomic status, teacher social support, and student reading literacy by cultural groups.

	African and Middle Eastern	Confucian	East-Central European	East European	English Speaking	Latin American	South East Asian	West European
A. Structural Equation Model Indices								
*χ*^2^(329), *p* < 0.001	17684.30	19487.92	27815.18	18625.21	28817.18	55013.75	95627.73	57445.07
Robust CFI	0.96	0.95	0.94	0.96	0.91	0.95	0.94	0.92
Robust TLI	0.95	0.94	0.93	0.95	0.90	0.94	0.91	0.91
Robust RMSEA (90% CI)	[0.04, 0.04]	[0.04, 0.04]	[0.04, 0.05]	[0.03, 0.04]	[0.03, 0.05]	[0.04, 0.04]	[0.05, 0.06]	[0.05, 0.06]
SRMR	0.05	0.05	0.04	0.04	0.06	0.05	0.05	0.05
B. Standardized Path Estimates								
SES	0.091*** (0.012)	0.234*** (0.014)	0.037*** (0.011)	0.314*** (0.010)	0.268*** (0.013)	0.185*** (0.010)	0.271** (0.013)	0.248*** (0.008)
TS	0.073*** (0.013)	0.148* (0.018)	0.005 (0.013)	−0.031 (0.019)	0.030 (0.029)	−0.005 (0.012)	0.062** (0.027)	0.005 (0.009)
TES	0.059*** (0.013)	0.028* (0.012)	0.070*** (0.013)	0.058*** (0.010)	0.111** (0.042)	0.144*** (0.013)	0.181*** (0.035)	0.141*** (0.020)
TF	0.058 (0.070)	0.099*** (0.012)	0.161*** (0.012)	0.050*** (0.011)	0.074* (0.037)	0.218* (0.102)	0.289* (0.140)	0.161*** (0.012)
SES*TS	−0.043*** (0.014)	0.017 (0.021)	0.016 (0.017)	−0.021 (0.014)	−0.035 (0.038)	−0.004 (0.010)	−0.040* (0.020)	−0.018 (0.014)
SES*TES	0.046*** (0.020)	0.050* (0.025)	0.062** (0.021)	0.049*** (0.016)	0.045** (0.026)	0.040*** (0.012)	0.096*** (0.027)	0.034** (0.018)
SES*TF	−0.051*** (0.020)	0.014 (0.023)	0.017 (0.014)	−0.005 (0.022)	0.063 (0.044)	−0.016 (0.012)	−0.107*** (0.031)	−0.024* (0.011)
*R* ^2^	0.22	0.15	0.24	0.22	0.15	0.28	0.35	0.18

CFI, Comparative Fit Index; TLI, Tucker–Lewis Index; RMSEA, Root Mean Squared Error of Approximation; SRMR, Standardized Root Mean Square Residual; SES, socioeconomic status; TS, teacher support; TES, teacher emotional support; TF, teacher feedback. Covariates were controlled for. *p < 0.05, **p < 0.01, ***p < 0.001.

**Table 5:** Summary of results across eight cultural groups.

	African and Middle Eastern	Confucian	East-Central European	East European	English Speaking	Latin American	South East Asian	West European
SES	+	+	+	+	+	+	+	+
TS	+	+					+	
TES	+	+	+	+	+	+	+	+
TF		+	+	+	+	+	+	+
SES*TS	−						−	
SES*TES	+	+	+	+	+	+	+	+
SES*TF	−						−	−

SES, socioeconomic status; TS, teacher support; TES, teacher emotional support; TF, teacher feedback. “+”: significantly positive relationship; “−”: significantly negative relationship; empty cell means non-significant relationship.

The bottom half of Table 4 presents the relationships between the variables of interest across different samples. A uniform positive effect of SES on reading literacy is identified across all eight cultures (0.037 < *β*s < 0.314, *p* < 0.001). TS indicates varied results, with a positive effect in African and Middle Easernt (*β* = 0.073, *p* < 0.001), Confucian (*β* = 0.148, *p* < 0.05), and South East Asian (*β* = 0.062, *p* < 0.01) cultures. No significant influence is noted in the other cultures. Next, all cultures consistently show a positive impact of TES on student achievement (0.028 < *β*s < 0.181, *p* < 0.05). TF reveals a positive effect throughout seven cultures (0.050 < *β*s < 0.218, *p* < 0.05), except for the African and Middle Eastern culture.

The SES-TS interaction shows negative influences in African and the Middle Eastern (*β* = −0.043, *p* < 0.001) and South East Asian (*β* = −0.040, *p* < 0.05) cultures, and no effect in other cultures. The interaction between SES and TES show positive effects on student achievement in all cultures (0.034 < *β*s < 0.096, *p* < 0.05). Lastly, for the interaction between SES and TF, a negative effect on student achievement is found in the African and the Middle Eastern (*β* = −0.051, *p* < 0.001), South East Asian (*β* = −0.107, *p* < 0.001), and West European (*β* = −0.024, *p* < 0.05) cultures. No significant effects were noted in the other cultures.

### 4.4 Multigroup SEM

Table 6 presents the results of the multigroup invariance tests for the key paths in the model. The fit indices indicate that all models demonstrate a good fit across cultural groups, with CFI > 0.90 and RMSEA < 0.08. In the baseline model (Model 1 in Table 6), all measurement parameters are constrained to equality across cultural groups, while the regression paths are freely estimated. Next, we sequentially imposed equality constraints on each path (Models 2–8 in Table 6) and compared them with the baseline model. Significant chi-square differences (Δ*χ*^2^) suggest variability in the magnitude of path coefficients across the eight cultures. For instance, in Model 2, the path from SES to reading literacy was constrained. A significant chi-square difference (Δ*χ*^2^ = 455.48, *p* < 0.001) indicated that the effect of SES on reading literacy is not consistent across cultural contexts.

**Table 6:** Multigroup invariance test of path coefficients.

Model	*χ* ^2^	CLI	TLI	RMSEA	SRMR	Δ*χ*^2^ (vs. model 1)
1. All paths free	13510.41	0.96	0.97	0.06	0.05	
2. SES → Reading literacy	13962.37	0.95	0.96	0.06	0.05	455.48***
3. TS → Reading Literacy	14532.25	0.95	0.96	0.07	0.06	574.96***
4. TES → Reading Literacy	14985.27	0.94	0.95	0.06	0.05	466.78***
5. TF → Reading literacy	15442.49	0.93	0.93	0.07	0.06	453.99***
6. SES * TS → Reading Literacy	15917.52	0.93	0.93	0.07	0.06	454.98***
7. SES * TES → Reading literacy	16268.71	0.92	0.92	0.08	0.07	355.57***
8. SES * TF → Reading literacy	16819.94	0.92	0.91	0.07	0.06	553.96***
9. All paths constrained	17364.37	0.92	0.91	0.08	0.07	954.87***

This table presents the results of the multigroup invariance test for path coefficients, examining the relationships between SES, Teacher Support (TS), Teacher Emotional Support (TES), Teacher Feedback (TF), and their interaction terms on reading literacy. In Model 1, all measurement parameters are constrained to equality across cultural groups, but the regression paths are freely estimated, serving as the baseline for comparison. In Models 2 through 8, one path at a time is constrained across groups to test for invariance. For instance, in Model 2, the path SES → Reading Literacy is constrained, meaning the effect of SES on Reading Literacy is held equal across all eight cultures. In Model 9, all paths (direct and interaction terms) are constrained across groups, providing a comprehensive test of invariance across all relationships. CFI, Comparative Fit Index; TLI, Tucker–Lewis Index; RMSEA, Root Mean Square Error of Approximation; SRMR, Standardized Root Mean Square Residual. A significant chi-square difference (Δ*χ*^2^) compared to the baseline model indicates that the strength of the specified relationship differs across cultural groups. ***p < 0.001.

To further explore these cultural differences, we conducted pairwise comparisons of the invariance tests between any two cultural groups. The results, presented in the Appendix Tables S2–S8, reveal varying patterns of similarities and differences in the strength of the relationships between variables. For example, Appendix Table S2 shows the results for the path from SES to reading literacy, highlighting significant differences between Western Europe and Confucian Countries (Δ*χ*^2^ = 12.42, *p* < 0.001).

## 5 Discussion

Utilizing Schwartz’s (2009) cultural value orientations, we categorized 70 countries/economies into eight distinct cultural groups. Using SEM on the PISA 2022 dataset, we examined the moderating effect of three TSS facets (TS, TES, and TF) on the relationship between SES and student reading literacy. We found that (1) SES is consistently positively related to reading literacy across eight cultures and (2) the TSS’s direct and moderate effects vary across different cultures. Below, we elucidate our findings from a cultural perspective.

### 5.1 SES effect

Consistent with prior studies, we found that SES has varying degrees of positive effects on reading literacy across eight cultures (Zhao et al., 2022). A “common-sense” mechanism underlying this relationship is that high-SES families are generally more equipped to invest in their children’s education. This investment can take various forms, such as providing educational resources (e.g., books and computers) or securing access to high-quality educational institutions (e.g., Conger and Donnellan, 2007), which contribute to enhanced academic outcomes. Additionally, high SES families often possess a more comprehensive understanding of the educational system and have greater expectations regarding the benefits of education (Cao et al., 2022). This, in turn, fosters a heightened learning motivation (Sohr-Preston et al., 2013).

### 5.2 TS effect

Our findings indicate that TS is positively correlated with reading literacy in three specific cultural contexts: African and Middle Eastern, Confucian, and South East Asian cultures. These regions share a common cultural characteristic, namely a pronounced emphasis on hierarchical structures coupled with the rejection of egalitarian principles (Figure 2; Schwartz, 2009). This hierarchical relationship imbues teacher roles with significant authority and respect and makes TS particularly impactful, as students in these cultures may be more receptive to guidance and teaching from figures of authority. This hierarchical structure also facilitates more streamlined communication between teachers and students, thereby making educational interventions more effective.

Moreover, contrary to CRT, we found that the interaction terms between SES and TS were negative in the African and Middle Eastern and South East Asian cultures, meaning that the relationship between SES and reading literacy was weaker for students who perceived high levels of TS. These two cultures exhibit higher levels of embeddedness than the rest of the world (Figure 2; Schwartz, 2009), underscoring the collective pursuit of communal objectives. These patterns can be better understood through the lens of teacher belief systems and culturally embedded pedagogical norms. In hierarchical cultures, where teachers are viewed as authoritative figures, providing structured guidance may be seen as a professional and moral responsibility (Hampden-Turner and Trompenaars, 1997). This aligns with collectivist values, where supporting disadvantaged students helps preserve group harmony and social cohesion (Yuan et al., 2024). In contrast, egalitarian cultures emphasize learner autonomy and equal treatment, and teachers may assume that students are equally capable of seeking help when needed (Schwartz, 2009). In addition, a thorough examination of the items revealed that TS pertains to perceptions of collective support (Bakchich et al., 2023). Generally, low SES students tend to prioritize interdependent values, whereas their high SES counterparts are generally oriented toward autonomy and individualism (Dittmann et al., 2020; Stephens et al., 2019). Consequently, low SES students are likely to be more receptive to forms of support that are collective or group-oriented, whereas high SES students may be more attuned to individualized and personal support.

### 5.3 TES effect

In accordance with a previous meta-analysis (Tao et al., 2022), our research demonstrates that TES is uniformly linked to enhanced reading literacy across eight cultures. This implies that an emotionally supportive atmosphere fostered by teachers contributes to elevated academic achievement among adolescents. Within the educational setting, students perceive this form of emotional support when they are treated equally and respectfully by their teachers, sense genuine warmth and unconditional positive regard from them, and feel that their ideas are actively listened to and considered (Calarco, 2014; Lareau, 2018).

In addition, interaction term reveals that the TSS positively moderates the SES-reading literacy relationship in all eight cultural contexts, suggesting that it amplifies the benefits of existing socioeconomic resources rather than compensating for their absence. Students from higher SES backgrounds often attend schools with more cultural capital, which aligns with the dominant culture of the educational system (Tramonte and Willms, 2010). When these students also receive high levels of TES, their existing cultural capital is likely to be validated and reinforced because their pre-existing cultural capital makes them more receptive to the forms of support that teachers are likely to offer, which are often based on identical dominant cultural norms (Lareau, 2018), such as self-expression and individual independence (Stephens et al., 2012). This pattern is consistent with Tsang and Lo (2025), who found that informal reading exposure (e.g., reading print or watching subtitled) is a key driver of vocabulary growth, while time spent on homework yields limited returns. TES may foster a motivational climate that also enhances students’ sense of relatedness, encouraging those who already possess strong self-confidence and access to informal learning opportunities to make fuller use of them (Lo et al., 2024), thereby reinforcing existing SES-based advantages.

### 5.4 TF effect

Consistent with extant literature (Wisniewski et al., 2020), our research corroborates that TF exhibits a positive correlation with reading literacy in seven of the eight investigated cultural contexts. Serving as an indispensable element of the educational dialog between teachers and students, teacher feedback is primarily geared toward assessing student performance or providing recommendations for improvement (Winstone and Carless, 2019). TF facilitates the reflective synthesis of knowledge by disseminating evaluative information on student performance (Gentrup et al., 2020). Delivering such constructive feedback empowers students to identify learning errors and make corrections, thereby enhancing their academic performance (Ma et al., 2022).

However, the TF effect is not statistically significant in the African and Middle Eastern cultural group. Several cultural and pedagogical considerations may explain this finding. From a cultural perspective, many countries in this group emphasize high power distance and embeddedness (Schwartz, 2009), where teacher authority is rarely questioned and feedback may be delivered in a more top-down, less dialogic manner (Archer and Sargeant, 2013). Students may perceive feedback as “final judgment” rather than constructive input for personal improvement, thereby limiting its motivational effect (To et al., 2025). From a pedagogical standpoint, TF in these settings may be more summative than formative, focusing on grades or rankings rather than actionable suggestions. In addition, such authority-centered feedback may fail to guide students, especially those from lower-SES backgrounds, toward productive exposure behaviors outside the classroom, such as engaging with accessible reading input. As Tsang and Lo (2025) argued, a formative, resource-linked feedback style that tells students what to read or watch next and how to work with it is more effective in promoting vocabulary development through informal exposure.

The moderation analysis suggests that the relationship between SES and reading literacy is weaker among students who reported a high perception of TF in the African and Middle Eastern, South East Asian, and Western European regions. Although these cultural groups have similar moderating effects, they may be rooted in distinct cultural values. Western European culture prioritizes egalitarianism, which presupposes that students are both capable and accountable for their actions (Schwartz, 2009). This egalitarian ethos may lead to a more uniform distribution of TF, irrespective of student SES backgrounds. Consequently, the impact of TF on reading literacy may be less pronounced among students who already perceive a high level of support, thereby attenuating the relationship between SES and reading literacy in these settings.

In contrast, societies in Africa, the Middle East, and South East Asia exhibit high levels of embeddedness, which underscores the collective pursuit of communal objectives (Schwartz, 2009). Given this focus on collectivism, it is conceivable that teachers may extend greater support to students who are socioeconomically disadvantaged (Wang et al., 2021). The aim of such support is to close the gap between these students and their more advantaged peers, leveling the playing field regarding educational outcomes (Condron, 2011).

### 5.5 Effect size

The effect sizes in this study were predominantly small. This could be attributed to the fact that SES and TSS are only two of the multiple factors influencing students’ reading literacy. Despite their small magnitudes, these effect sizes align with previous empirical work on cultural capital theory and the role of TSS. While many scholars have traditionally relied on Cohen’s (2013) guidelines to interpret the significance of effect sizes, the recent literature suggests a more nuanced approach. Specifically, meta-analyses suggest that smaller effect sizes are more prevalent in the social sciences and education fields (Hattie, 2008).

Moreover, the effect sizes varied across the eight cultural groups, which could have partially contributed to cultural values. For example, East-Central European countries have lower SES for reading achievement associations (i.e., regression) among the eight cultures. This finding aligns well with the cultural group’s high emphasis on harmony and intellectual autonomy and lower emphasis on hierarchy (Schwartz, 2009).

### 5.6 Cultural values

One salient contribution of this study is its incorporation of Schwartz’s (2009) cultural value orientation as a framework for understanding the interactions between SES, three TSS facets, and reading literacy across diverse cultural contexts. By categorizing countries/economies into eight distinct cultural clusters, we elucidate the extent to which specific cultural value orientations moderate the aforementioned relationships. This approach enables a more targeted understanding of how cultural values (e.g., hierarchy, egalitarianism, and embeddedness) shape educational outcomes through their interaction with SES and TSS mechanisms. Therefore, this study provides educational policymakers and practitioners with culturally contextualized insights that can inform more effective and culturally sensitive educational interventions.

### 5.7 Revisiting the research questions

Regarding RQ1, we found that all three facets of TSS—general teacher support (TS), emotional support (TES), and feedback (TF)—had positive associations with reading literacy, though their effects varied across cultural groups. TES showed the most consistent and robust effect across all eight cultures, followed by TF, while TS had significant effects in only three cultural clusters (African and Middle Eastern, Confucian, and South East Asian). These findings suggest that different forms of support may resonate more strongly in certain cultural settings, shaped by value orientations such as hierarchy, collectivism, and power distance.

In addressing RQ2, we observed that TSS moderates the SES–reading literacy relationship in culturally specific ways. Notably, the interaction between SES and TS was negative in more collectivist or embedded cultures, suggesting a compensatory effect favoring low-SES students. Conversely, the interaction between SES and TES was positive across all cultures, indicating that emotional support tends to amplify existing SES-based advantages. The interaction between SES and TF revealed a more nuanced pattern: in some hierarchical or egalitarian cultures, teacher feedback appeared to buffer the SES gap, while in others it had no significant effect. These patterns highlight the complex and context-dependent role of TSS in shaping educational equity. Together, these findings underscore the importance of culturally responsive pedagogies and the need for localized interpretations of teacher-student dynamics in international educational research.

### 5.8 Limitations and future research

Our study has several limitations. First, SES is a multifacet construct, typically comprising parental education, household income, and occupational status (Harris, 2023). In this study, we used the composite SES index provided by PISA, which integrates these elements into a single latent variable. While this simplifies modeling, it may mask the unique effects of individual components. For instance, parental education may shape reading literacy differently than income. Future research could explore how individual facets of SES interact with TSS and influence reading literacy.

Next, although this study focused on social support from teachers, future research could benefit from exploring the role of social support from parents and peers (Tsang and Lo, 2025). Third, TSS was measured through student self-reports. Such data may be subject to perceptual bias and influenced by cultural response styles. Although measurement invariance was tested, cross-cultural differences in how students perceive and interpret support may still affect comparability. Future research should consider multi-informant or observational data to validate and extend the findings.

Fourth, while our discussion focused on cultural explanations for the negative interaction terms, alternative interpretations should also be considered. One such possibility is differential item functioning (DIF), wherein students with different SES backgrounds may interpret and respond to TSS items in systematically different ways, even if they experience similar levels of support. For example, high-SES students may view certain forms of support as redundant or less meaningful, while low-SES students may perceive the same support as highly impactful. Such perception differences can introduce measurement bias, potentially distorting the estimated interaction effects. Therefore, negative interactions between SES and TSS might partly reflect psychometric artifacts rather than genuine differences in educational effectiveness.

Fifth, our study adopted a design-based approach to variance estimation, incorporating replicate weights and a pooled within-cluster covariance matrix to account for the hierarchical sampling structure. While this method adequately adjusts for clustering and complex sampling, it represents one of several possible strategies. For instance, using cluster-robust standard errors at the school level is theoretically feasible and may offer alternative insights into the robustness of parameter estimates. However, to avoid confounding between variance estimation approaches, we did not apply multiple methods simultaneously. Future research could conduct sensitivity analyses using different variance estimation strategies to further assess the stability of findings.

Finally, the generalizability of our findings is limited by the secondary analysis of the PISA dataset. Although the PISA has an expanding number of participating economies, there are other countries within the eight cultural regions that did not participate. Expectancy-Value Theory (EVT) accounts for variations in culture and individual differences, including a broader range of countries in empirical testing, and can provide more insight into the impact of cultural values on student-related variables.

### 5.9 Implications

This study has several implications. Our findings indicate that all three TSS facets are positively associated with students’ reading literacy across the eight cultures. This suggests that the role of TSS is not merely a localized phenomenon but a fundamental aspect of effective education (An et al., 2022; Guo and Wang, 2021; Tao et al., 2022). Thus, this study emphasizes the necessity for educators to foster supportive relationships with students as a means of enhancing reading literacy, irrespective of the cultural background. Although the observed effect sizes were generally small, their consistency across diverse cultural contexts highlights their practical relevance. In large-scale education systems, even small effects can translate into meaningful improvements when implemented across numerous classrooms and schools (Funder and Ozer, 2019). For policymakers, this highlights the importance of supporting scalable interventions such as teacher training programs focused on social–emotional support. While individual gains may be modest, the cumulative impact of these initiatives at a national or system level can be substantial.

Moreover, our study indicates that the moderating effects of the three TSS facets on the SES-reading achievement link exhibit cultural variation, explaining the disparate findings of previous studies on TSS. The inconsistency in prior studies might arise from the fact that they involved participants from diverse countries or cultural backgrounds. However, the focus on different TSS facets could also account for the varying results. For example, a researcher examining data primarily from Western European regions and focusing mainly on the TES domain might derive different conclusions about the moderating effect of TSS than researchers who concentrate on the TF domain (Bakchich et al., 2023).

Finally, integrating Schwartz’s (2009) cultural value orientation enriches our understanding of the relationships among SES, TSS, and reading literacy. Our results offer culturally specific recommendations regarding the three TSS facets. TS was particularly effective in African and Middle Eastern, Confucian, and South East Asian cultures, where hierarchical values prevail. In these contexts, teacher authority is highly respected, and strengthening teacher–student relationships can be especially impactful. Therefore, training programs in these regions should emphasize building respectful and supportive classroom dynamics. Teachers may scaffold weekly independent-learning routines and explicitly curate low-cost exposure opportunities (e.g., reading, listening, or subtitled input), gradually fading support to promote self-regulation. TES showed consistent positive effects across all eight cultures, making it a universally valuable component of teacher practice regardless of cultural background. Thus, TES can be used to normalize academic struggle and cultivate students’ growth mindset and universal teacher training programs should thus embed emotional support strategies as core competencies. However, as TES tend to boost the SES-based advantages on reading literacy, unless deliberately targeted, such benefits may accrue disproportionately to students already endowed with greater capital. TF was effective in most cultural groups but showed limited impact in African and Middle Eastern contexts. This suggests that in some regions, feedback may need to be adapted to align with students’ cultural expectations or communication norms. Together, consistent with longitudinal evidence from Hong Kong primary learners (Tsang and Lo, 2025), general informal exposure shows small but meaningful associations with socioeconomic background and predicts vocabulary growth, a core lever for reading literacy. We therefore interpret culture-contingent moderations of teacher social support as partly operating through their differential capacity to cultivate independent-learning habits and steer students into effective, low-cost exposure outside school.

This signals to policymakers and educators that a one-size-fits-all approach to educational interventions may be ineffective because of the heterogeneity of cultural values across different societies. It also suggests that even small effects of TSS may be amplified or diminished depending on the cultural alignment of educational practices with prevailing value systems. In particular, the efficacy of TS across cultures requires culturally adaptive teaching strategies. This study invites future research to treat culture as a dynamic construct with multiple value dimensions, which could lead to more effective and culturally inclusive educational policies and interventions, thereby enhancing their relevance and impact across various sociocultural landscapes (Haw and King, 2022).

## Data Availability

The original contributions presented in the study are included in the article/Supplementary material, further inquiries can be directed to the corresponding author.

## Footnotes

^1^We accounted for the complex survey design of PISA 2022 by incorporating the final student weight (W_FSTUWT) and the 80 BRR replicate weights (W_FSTURWT1–W_FSTURWT80) with Fay’s adjustment (*ρ* = 0.5), implemented via the survey and lavaan.survey packages in R.
