# Supplementary material for: Beyond socioeconomic status: the cross-cultural interplay of perceived teacher social support in reading literacy
Source: Front Psychol. 2025 Nov 11;16:1642504. doi: 10.3389/fpsyg.2025.1642504 (PMC12643996; doi:10.3389/fpsyg.2025.1642504)
Supplement: Supplementary file 1 [file Supplementary_file_1.docx]

Appendix

Table S1. Loadings and Reliability of Teacher Social Support Facets Across Cultures

| \| Cultural Group \| Teacher Support \| \| \| Teacher Emotional Support \| \| \| Teacher Feedback \| \| \| \| --- \| --- \| --- \| --- \| --- \| --- \| --- \| --- \| --- \| --- \| \| Item loading \| α \| ω \| Item loading \| α \| ω \| Item loading \| α \| ω \| \| African & Middle Eastern \| .62–.88 \| 0.84 \| 0.86 \| .58–.85 \| 0.8 \| 0.83 \| .57–.83 \| 0.79 \| 0.81 \| \| Confucian \| .59–.84 \| 0.79 \| 0.81 \| .60–.87 \| 0.77 \| 0.79 \| .54–.80 \| 0.75 \| 0.77 \| \| East-Central European \| .64–.87 \| 0.85 \| 0.88 \| .59–.84 \| 0.82 \| 0.84 \| .60–.85 \| 0.81 \| 0.83 \| \| East European \| .60–.85 \| 0.82 \| 0.84 \| .55–.82 \| 0.75 \| 0.78 \| .52–.78 \| 0.72 \| 0.75 \| \| English Speaking \| .63–.88 \| 0.83 \| 0.85 \| .63–.86 \| 0.81 \| 0.83 \| .61–.84 \| 0.8 \| 0.82 \| \| Latin American \| .58–.81 \| 0.76 \| 0.79 \| .61–.85 \| 0.79 \| 0.82 \| .59–.83 \| 0.78 \| 0.8 \| \| South East Asian \| .59–.80 \| 0.74 \| 0.77 \| .56–.80 \| 0.73 \| 0.76 \| .53–.76 \| 0.7 \| 0.74 \| \| West European \| .65–.88 \| 0.86 \| 0.89 \| .60–.86 \| 0.84 \| 0.86 \| .62–.87 \| 0.85 \| 0.87 \| |  |  |  |  |  |  |  |  |
| --- | --- | --- | --- | --- | --- | --- | --- | --- | --- | --- | --- | --- | --- | --- | --- | --- | --- | --- | --- | --- | --- | --- | --- | --- | --- | --- | --- | --- | --- | --- | --- | --- | --- | --- | --- | --- | --- | --- | --- | --- | --- | --- | --- | --- | --- | --- | --- | --- | --- | --- | --- | --- | --- | --- | --- | --- | --- | --- | --- | --- | --- | --- | --- | --- | --- | --- | --- | --- | --- | --- | --- | --- | --- | --- | --- | --- | --- | --- | --- | --- | --- | --- | --- | --- | --- | --- | --- | --- | --- | --- | --- | --- | --- | --- | --- | --- | --- | --- | --- | --- | --- | --- | --- | --- | --- | --- | --- |
| *Note*: Teacher Support, Teacher Emotional Support, and Teacher Feedback were measured using four, three, and three items, respectively. Cronbach’s α and McDonald’s ω represent internal consistency reliability within each cultural cluster. |  |  |  |  |  |  |  |  |
|  |  |  |  |  |  |  |  |  |
|  |  |  |  |  |  |  |  |  |
|  |  |  |  |  |  |  |  |  |
|  |  |  |  |  |  |  |  |  |
|  |  |  |  |  |  |  |  |  |
|  |  |  |  |  |  |  |  |  |
|  |  |  |  |  |  |  |  |  |
|  |  |  |  |  |  |  |  |  |

Table S1: SES → Reading Literacy Regression Invariance Test by Cultural Groups (Δχ²)

| Cultural Group | Western Europe | East Central Europe | East Europe | Latin America | English Speaking | Confucian Countries | South East Asia |
| --- | --- | --- | --- | --- | --- | --- | --- |
| Western Europe |  |  |  |  |  |  |  |
| East Central Europe | 3.53* |  |  |  |  |  |  |
| East Europe | 2.13 | 4.73* |  |  |  |  |  |
| Latin America | 10.23*** | 6.35* | 7.89** |  |  |  |  |
| English Speaking | 1.42 | 3.31 | 1.68 | 4.56* |  |  |  |
| Confucian Countries | 12.42*** | 9.12** | 8.46** | 15.25*** | 8.97** |  |  |
| South East Asia | 3.87* | 5.62** | 2.45 | 11.84*** | 4.77* | 7.85** |  |
| Africa & Middle East | 28.43*** | 24.53*** | 27.79*** | 13.52** | 15.24*** | 33.46*** | 18.98*** |

*Note*: Table examines the SES → Reading Literacy path across different cultural groups. A significant Δχ² means the impact of SES on reading literacy varies significantly across the compared two regions. * *p* < 0.05; ** *p* < 0.01; *** *p* < 0.001.

Table S2: TS → Reading Literacy Regression Invariance Test by Cultural Group (Δχ²)

| cultural Group | Western Europe | East Central Europe | East Europe | Latin America | English Speaking | Confucian Countries | South East Asia |
| --- | --- | --- | --- | --- | --- | --- | --- |
| Western Europe |  |  |  |  |  |  |  |
| East Central Europe | 4.12* |  |  |  |  |  |  |
| East Europe | 2.19 | 3.06 |  |  |  |  |  |
| Latin America | 8.33** | 6.21* | 5.67* |  |  |  |  |
| English Speaking | 0.56 | 0.82 | 0.65 | 1.38 |  |  |  |
| Confucian Countries | 12.47*** | 10.13** | 8.45** | 20.37*** | 7.82** |  |  |
| South East Asia | 2.29 | 3.44* | 1.54 | 14.35*** | 2.98* | 6.85** |  |
| Africa & Middle East | 33.53*** | 26.86*** | 30.48*** | 9.13** | 10.91*** | 42.13*** | 19.88*** |

*Note*: Table examines the Teacher support (TS) → Reading Literacy path across different cultural groups. A significant Δχ² means the impact of TS on reading literacy varies significantly across the compared two regions. * *p* < 0.05; ** *p* < 0.01; *** *p* < 0.001.

Table S3: TES → Reading Literacy Regression Invariance Test by Cultural Group (Δχ²)

| Cultural Group | Western Europe | East Central Europe | East Europe | Latin America | English Speaking | Confucian Countries | South East Asia |
| --- | --- | --- | --- | --- | --- | --- | --- |
| Western Europe |  |  |  |  |  |  |  |
| East Central Europe | 3.72* |  |  |  |  |  |  |
| East Europe | 1.45 | 4.64* |  |  |  |  |  |
| Latin America | 10.27*** | 8.34** | 6.14** |  |  |  |  |
| English Speaking | 0.99 | 1.76 | 1.04 | 2.57* |  |  |  |
| Confucian Countries | 11.26*** | 9.71** | 7.44** | 18.96*** | 6.91** |  |  |
| South East Asia | 1.18 | 4.85** | 0.90 | 12.33*** | 3.97* | 5.84** |  |
| Africa & Middle East | 31.49*** | 27.42*** | 29.53*** | 8.32** | 9.91*** | 43.32*** | 17.44*** |

*Note*: Table examines the Teacher emotional support (TES) → Reading Literacy path across different cultural groups. A significant Δχ² means the impact of TES on reading literacy varies significantly across the compared two regions. * *p* < 0.05; ** *p* < 0.01; *** *p* < 0.001.

Table S4: TF → Reading Literacy Regression Invariance Test by Cultural Group (Δχ²)

| Cultural Group | Western Europe | East Central Europe | East Europe | Latin America | English Speaking | Confucian Countries | South East Asia |
| --- | --- | --- | --- | --- | --- | --- | --- |
| Western Europe |  |  |  |  |  |  |  |
| East Central Europe | 2.42 |  |  |  |  |  |  |
| East Europe | 3.76** | 5.12** |  |  |  |  |  |
| Latin America | 11.25*** | 9.97*** | 7.15** |  |  |  |  |
| English Speaking | 1.24 | 1.94 | 1.27 | 3.16* |  |  |  |
| Confucian Countries | 13.17*** | 10.78*** | 8.47** | 22.99*** | 7.83** |  |  |
| South East Asia | 2.66* | 3.76** | 1.66 | 13.57*** | 2.84* | 7.66** |  |
| Africa & Middle East | 35.13*** | 29.44*** | 32.27*** | 10.48** | 12.35*** | 44.15*** | 18.74*** |

*Note*: Table examines the teacher feedback (TF) → Reading Literacy path across different cultural groups. A significant Δχ² means the impact of TF on reading literacy varies significantly across the compared two regions. * *p* < 0.05; ** *p* < 0.01; *** *p* < 0.001.

Table S5. SES * TS → Reading Literacy Regression Invariance Test by Cultural Group (Δχ²)

| Cultural Group | Western Europe | East Central Europe | East Europe | Latin America | English Speaking | Confucian Countries | South East Asia |
| --- | --- | --- | --- | --- | --- | --- | --- |
| Western Europe |  |  |  |  |  |  |  |
| East Central Europe | 2.15 |  |  |  |  |  |  |
| East Europe | 3.64** | 4.85** |  |  |  |  |  |
| Latin America | 9.97*** | 7.25** | 6.13** |  |  |  |  |
| English Speaking | 0.86 | 1.66 | 0.87 | 3.87* |  |  |  |
| Confucian Countries | 14.13*** | 9.78** | 8.14** | 19.58*** | 8.45** |  |  |
| South East Asia | 2.78* | 3.45* | 1.48 | 15.45*** | 1.47 | 6.53** |  |
| Africa & Middle East | 29.45*** | 28.24*** | 33.59*** | 12.14** | 13.54*** | 41.87*** | 20.12*** |

*Note*. Table examines the SES*Teacher Support (TS) → Reading Literacy path across different cultural groups. A significant Δχ² means the impact of SES*TS on reading literacy varies significantly across the compared two regions. * *p* < 0.05; ** *p* < 0.01; *** *p* < 0.001.

Table S6. SES * TES → Reading Literacy Regression Invariance Test by Cultural Group (Δχ²)

| Cultural Group | Western Europe | East Central Europe | East Europe | Latin America | English Speaking | Confucian Countries | South East Asia |
| --- | --- | --- | --- | --- | --- | --- | --- |
| Western Europe |  |  |  |  |  |  |  |
| East Central Europe | 2.45 |  |  |  |  |  |  |
| East Europe | 1.48 | 3.35* |  |  |  |  |  |
| Latin America | 8.39** | 5.27* | 6.15** |  |  |  |  |
| English Speaking | 1.37 | 2.59 | 0.97 | 3.97* |  |  |  |
| Confucian Countries | 9.35*** | 7.12** | 8.46** | 13.34*** | 7.87** |  |  |
| South East Asia | 1.13 | 4.37* | 1.54 | 9.80** | 3.31* | 5.74** |  |
| Africa & Middle East | 26.46*** | 22.23*** | 28.46*** | 10.57** | 12.43*** | 33.12*** | 17.81*** |

*Note*. Table examines the SES*Teacher Emotional Support (TES) → Reading Literacy path across different cultural groups. A significant Δχ² means the impact of SES*TES on reading literacy varies significantly across the compared two regions. * *p* < 0.05; ** *p* < 0.01; *** *p* < 0.001.

Table S7. SES * TF → Reading Literacy Regression Invariance Test by Cultural Group (Δχ²)

| Cultural Group | Western Europe | East Central Europe | East Europe | Latin America | English Speaking | Confucian Countries | South East Asia |
| --- | --- | --- | --- | --- | --- | --- | --- |
| Western Europe |  |  |  |  |  |  |  |
| East Central Europe | 3.41* |  |  |  |  |  |  |
| East Europe | 2.12 | 4.13* |  |  |  |  |  |
| Latin America | 9.27** | 6.34* | 7.48** |  |  |  |  |
| English Speaking | 1.20 | 2.89 | 1.46 | 3.01 |  |  |  |
| Confucian Countries | 11.14*** | 8.47** | 7.54** | 14.32*** | 8.57** |  |  |
| South East Asia | 2.23 | 5.76** | 2.87 | 10.89*** | 4.36* | 6.97** |  |
| Africa & Middle East | 30.47*** | 25.51*** | 29.41*** | 12.34** | 14.23*** | 35.12*** | 18.70*** |

*Note*. Table examines the SES* Teacher feedback (TF) → Reading Literacy path across different cultural groups. A significant Δχ² means the impact of SES*TF on reading literacy varies significantly across the compared two regions. * *p* < 0.05; ** *p* < 0.01; *** *p* < 0.001.

Note for partial scalar invariance at Table 3.

To achieve partial scalar invariance for the Teacher Support (TS) construct, we released the intercepts of two items:

(1) “The teacher shows an interest in every student's learning”

(2) “The teacher gives extra help when students need it”

These items were likely culturally variant due to differences in educational norms and teacher–student interaction expectations. Specifically, the concept of a teacher “showing interest” in every student may not be universally expected or valued in all educational contexts, particularly in high power-distance cultures. Similarly, the provision of “extra help” may vary depending on institutional resources, student–teacher dynamics, and cultural norms around help-seeking and academic support.

Furthermore, we released the intercept of TES item (“I felt that my teacher understood me”). This item was likely culturally variant due to its highly subjective and relational nature, which is interpreted differently across cultural contexts. In some collectivist or high-context cultures, emotional understanding by authority figures may be implicit or even discouraged, whereas in individualistic cultures, emotional attunement is often expected and explicitly communicated. Additionally, the translation of “understood me” into local languages may introduce semantic nuances that alter the psychological meaning of the item.
